# The ameliorative effect of curcumin extract on the morphological and skeletal abnormalities induced by sunset yellow and tartrazine in the developing chick embryo *Gallus domesticus*

**DOI:** 10.1016/j.heliyon.2020.e03305

**Published:** 2020-01-31

**Authors:** Hend T. El-Borm, Gamal M. Badawy, Sobhy H. El-Nabi, Wessam A. El-Sherif, Marwa N. Atallah

**Affiliations:** a Vertebrates, Comparative Anatomy and Embryology– Zoology Department, Faculty of Science, Menoufia University, Egypt; b Experimental Embryology- Zoology Department, Faculty of Science, Menoufia University, Egypt; c Molecular Biology- Zoology Department, Faculty of Science, Menoufia University, Egypt; d Zoology Department, Faculty of Science, Menoufia University, Egypt

**Keywords:** Food science, Biological sciences, Developmental biology, Embryology, Health sciences, Chick embryo, Sunset yellow, Tartrazine, Curcumin, Malformation, Skeletal, Morphological

## Abstract

Previous studies have suggested that food dyes are responsible for causing number of health problems. The study under consideration aims to show the possible morphological and skeletal malformation induced due to *in ovo* administration of sunset yellow (SY) and tartrazine (Tz) with or without curcumin (Cur) during organogenesis of developing chick embryo at doses 1.575mg/egg, 0.375mg/egg and 3mg/kg eggs for SY, Tz and Cur comparing with control. The investigation revealed evident reduction in the weight and length of embryos as well as malformations in feather, head, and limbs. Most of the congenital malformations were seen in SY and Tz injected groups such as short beak, excencephaly, kniked tail and pygostyle, curved scapula and retardation in the degree of ossification were the most evident in endoskeleton malformation. In addition, the length of ossified long bones in SY and Tz groups was affected. Co-administration of Cur with SY and Tz ameliorate the reversed effect of SY and Tz on the shape, length, body weight and skeleton of embryos.

## 1 Introduction

Food additives are products added to the food stuffs with an aim of improving its, flavor, taste, color, texture and conservation (Tawfek et al., 2015). They are labeled with a code consists of the letter E and specific number to be identified all over the world (Saltmarsh and Insall, 2013; Carocho et al., 2014; Abdel Ghany, 2015) but some countries use only the number (Inetianbor et al., 2015). Although food additives are used to make bad quality food looks good they can cause a number of health problems like diarrhea, skin irritation, stomach disorders, vomiting or an increase in the body heat (Amin and Al-Shehri, 2018). In addition, recent study by El-Borm et al. (2019) has been shown that food colorants caused many histopathological effects on liver and kidney of the chick embryo. Colorants are large category of food additive used in food, drugs and cosmetics after agreement of Food and Drug Administration (FDA) (Pressman et al., 2017). In Egypt there is a sharp increase in the use of synthetic color especially in the rural area, due to their low cost and stability (Abd Elhalem et al., 2016; Ali et al., 2016).

Curcumin (Cur) is a polyphenolic natural food color found in Turmeric (*curcuma longa*) used as a spice and has vital role in both medical and scientific field. It is widely used as food coloring pigment and preservative. The major constituents of turmeric roots are the volatile oils and the curcuminoids (ie., curcumin, demethoxycurcumin, and bisdemethoxycurcumin). Typical curcumin extracts available commercially contain about 75% curcumin, 20% demethoxycurcumin, and 5% bisdemethoxycurcumin, all of which are thought to be biologically active and possess protective properties (Pulido-Moran et al., 2016). The Joint FAO/WHO Expert Committee on Food Additives (JECFA) allocated an acceptable daily intake (ADI) of Cur is 0–3 mg/kg bw/day (EFSA, 2010; Amalraj et al., 2017). FDA have been proved curcuminoids as" Generally Recognized as Safe" (GRAS) when used in a suitable dose (Rodrigues et al., 2015; Hewlings and Kalman, 2017). Cur has several therapeutic properties such as antioxidant (Alsamydai and Jaber, 2018). *In vivo* and *in vitro* studies reported that Cur has a protective against oxidative stress (Menon and Sudheer, 2007). It has the ability of scavenging different forms of free radicals. Also, Cur's ability of capturing hydrogen peroxide is higher than that of the commercial antioxidants at the same concentration (Ismail and Sakr, 2016; Hewlings and Kalman, 2017; Jovičić et al., 2017). The clinical studies proved efficiency of Cur in the repair of tissues in a complex process that involves inflammation, granulation, and remodeling of the tissue (Alsamydai and Jaber, 2018). Ozaki et al. (2000) reported that Cur is a potent stimulator of osteoclast apoptosis. Turmeric extracts prevented the activation of the transcription factor NF-κB and suppressed the subsequent expression of genes mediating the destruction of cartilage and bone, including RANK-L, the ligand that binds to and activates the receptor activator for NF-κB (RANK) on osteoclast precursors, which is a necessary and sufficient stimulus for the differentiation of bone-destroying osteoclasts (Wright et al., 2010; Badawy et al., 2018). Turmeric has been used during pregnancy in a variety of traditional medical systems for thousand years and no Western authority recommends against its use during pregnancy (Asher and Spelman, 2013).

Sunset yellow (SY) (E110) is a synthetic coal tar and mono azo yellow dye derived from petroleum aromatic hydrocarbons, commonly used in food industry especially fermented foods which must be heat treated (Kushwah and Bharti, 2013; Saatloo et al., 2015) also, in cosmetics and pharmaceutical field (Allam & Kumar, 2011). It's alternative to natural color known as carminic acid (Carocho et al., 2014; Masone and Chanforan, 2015; Abd Elhalem et al., 2016; Rovina et al., 2017). Although using SY in food industry, it has adverse effect on human health due to the presence of aromatic ring and azo function group which has toxic derivatives formed during the dye degradation process (Carocho et al., 2014; Khayyat et al., 2018).

Tartrazine (Tz) (E102) is an artificial lemon yellow azo dye derived from coal tar. It is water soluble mono azo color which used in human food, pharmaceutical products and cosmetics. Many countries are using Tz as saffron substitute (Himri et al., 2011; Ali et al., 2016; Khayyat et al., 2017).The JECFA evaluated ADI to be 0–7.5 mg/kg/day (Moutinho et al., 2007; Himri et al., 2011; Ali et al., 2016)**.** The metabolites generate ROS which cause oxidative stress (EFSA, 2009; Himri et al., 2011; Khayyat et al., 2017). Also, it promoting lipid peroxidation and inhibiting endogenous antioxidant defense enzymes which, in turn, accelerate oxidative stress and damage most cellular components leading finally to cell death (Morales et al., 2004; Boussada et al., 2017).

The present study was planned to illustrate the possible morphological and skeletal malformations induced by three food coloring agents (natural and synthetic) on the development of chick embryo and the anticipated role of natural color against the toxicity induced by synthetic coloring agents.

## 2 Materials and methods

### 2.1 Egg incubation and grouping

Principles of animal care and use were carefully followed during conducting the present study according to the guide for the care and use of laboratory animals approved by faculty of science, Menoufia university, Egypt (Approval No. MNSE2190) and according to the National Institutes of Health guide for the care and use of laboratory animals (NIH publications No. 8023, received 1978). Fresh fertilized chicken eggs (*Gallus domesticus*) were obtained from a local hatchery at Menouf, Menoufia governorate. Before incubation at 37 °C in an artificial incubator, eggs were cleaned with distilled water followed by 70% ethanol weighted (50 ± 5 g) and then labeled. To ensure the relevant humidity (65%), an open 1-liter container filled with distilled water was placed at the bottom of the incubator. The eggs were put horizontally and turned over, at least three times a day. Eggs were candled before treatment and the unfertilized eggs were excluded and the remaining eggs were divided into seven groups (40 eggs each) and injected at the six day of incubation with a single dose which is equivalent to14 times ADI for synthetic colorants SY and Tz. A total of 280 fertilized eggs were included.

1 Group A was not subjected to any injection (Control group).
2 Group B was injected in *ovo* with 0.2 ml of sterile water (Sham group).
3 Group C was injected in *ovo* with 0.2 ml of Cur extract at a dose of 3 mg/kg eggs (Amalraj et al., 2017).
4 Group D was injected in *ovo* with 0.2 ml of SY at a dose of 1.575 mg/egg (ADI) (Hashem et al., 2011).
5 Group E was injected in *ovo* with 0.2 ml of Tz at a dose of 0.375 mg/egg (ADI) (Moutinho et al., 2007; JECFA, 2016).
6 Group F was injected in *ovo* with 0.2 ml of 1:1 mixture of Cur extract and SY at the same doses for both.
7 Group G was injected in *ovo* with 0.2 ml of 1:1 mixture of Cur extract and Tz at the same doses for both.

### 2.2 Coloring agents' administration

Cur, as a natural food coloring agent was obtained from a local herbal store while SY FCF(E110) and Tz FD&C yellow n°5 (E102) were obtained in a pure powder form, from Kamina Co. (cairo, Egypt). All coloring agents were dissolved (SY and Tz) or suspended (Cur) in sterile water and injected in *ovo* in 0.2 ml/egg.

#### 2.2.1 Water extraction of Cur

Dry rhizomes of the plant (*Curcuma longa*) were bought from a herbal store at Shebeen El-Koom, Menoufia, Egypt and crushed into powder. 125g of the powder were macerated in 1000 ml of sterile water for 12 h at room temperature and filtered through a 5 μm filter paper. The concentration of obtained extract was 24 mg/ml (Kamtchouing et al., 2002). Cur extract was applied at a dose of 3 mg/kg eggs (Amalraj et al., 2017; JECFA, 2016). At the 6^th^ day of incubation and using a sterile syringe, 0.2 ml of fluid was injected as single dose into the air sac. The holes were carefully sealed with molten paraffin wax.

#### 2.2.1 Embryo collection

At the 20^th^ day of incubation, the egg shells were broken with a scalpel and embryos were carefully freed from the egg shell. The living embryos were examined carefully for investigating the morphological abnormalities before photographing. Embryo-toxicity were determined at first by counting the number of living and dead embryos and morphometric parameters were recorded.

### 2.3 Investigated parameters

#### 2.3.1 Morphometric parameters

The body weights (gm) as well as the Crown-rump length (cm) of embryos of both control and experimental groups were recorded.

#### 2.3.2 Endo-skeletal investigation

For endo-skeletal preparation, double staining transparency technique was applied using the chonrogenic indicator Alcian blue and osteogenic indicator Alizarin red S for staining cartilage and bone, respectively. This has been achieved following a method originally introduced by Cortés-Delgado et al. (2009) and modified by Badawy et al. (2012). Specimens were then examined in glycerol at low resolution under Heerbrugg M3C dissection microscope and at high resolution using a Letiz Laborlux S light microscope. photographs of representative samples were taken using Sony digital camera. Lengths of long bones *i.e*. Humerus, radius, ulna, femur, tibia and fibula were measured in all chick embryos.

#### 2.3.3 Data evaluation and statistical analysis

All data sets were expressed as mean ± standard error of the mean (SEM). The data were analyzed statistically for normal distribution (student's T test) and homogeneity of variances (Levene test) using statistical package of social sciences (IBM SPSS) statistics software for windows, Version22 (IBM corp., Armonk, NY,USA). Differences were considered insignificant whenever P > 0.05. The significances of the obtained data were classified into three categories, i.e. P < 0.0001, P < 0.001 and P < 0.05 according to the obtained P values.

## 3 Results

### 3.1 Mortality rate

The mortality rate was represented by dead embryos either normally or due to exposure to food coloring agent at the end of experimentation. The ratio of both dead and living embryos was investigated and their number was recorded in Table 1. There was a relatively low mortality among control, sham and experimental group in *ovo* injected with Cur during the organogenesis phase of incubation. On the other hand, the mortality of embryos in *ovo* injected with SY and Tz in the same period was highly increased compared with control. Co- administration of Cur with SY or Tz ameliorated the ratio of dead embryos in both groups.

**Table 1:** Mortality rate of chick embryos (%) recorded in the 20^th^ day of incubation in different groups at the end of experimentation.

Groups	No of eggs used	No of surviving embryos	Mortality (%)
Cont	40	36	10%
Sh	40	34	15%
Cur	40	30	25%
SY	40	14	65%
Tz	40	20	50%
SY+ Cur	40	22	45%
Tz +Cur	40	26	35%

### 3.2 Morphological observations

**Figure 1:**
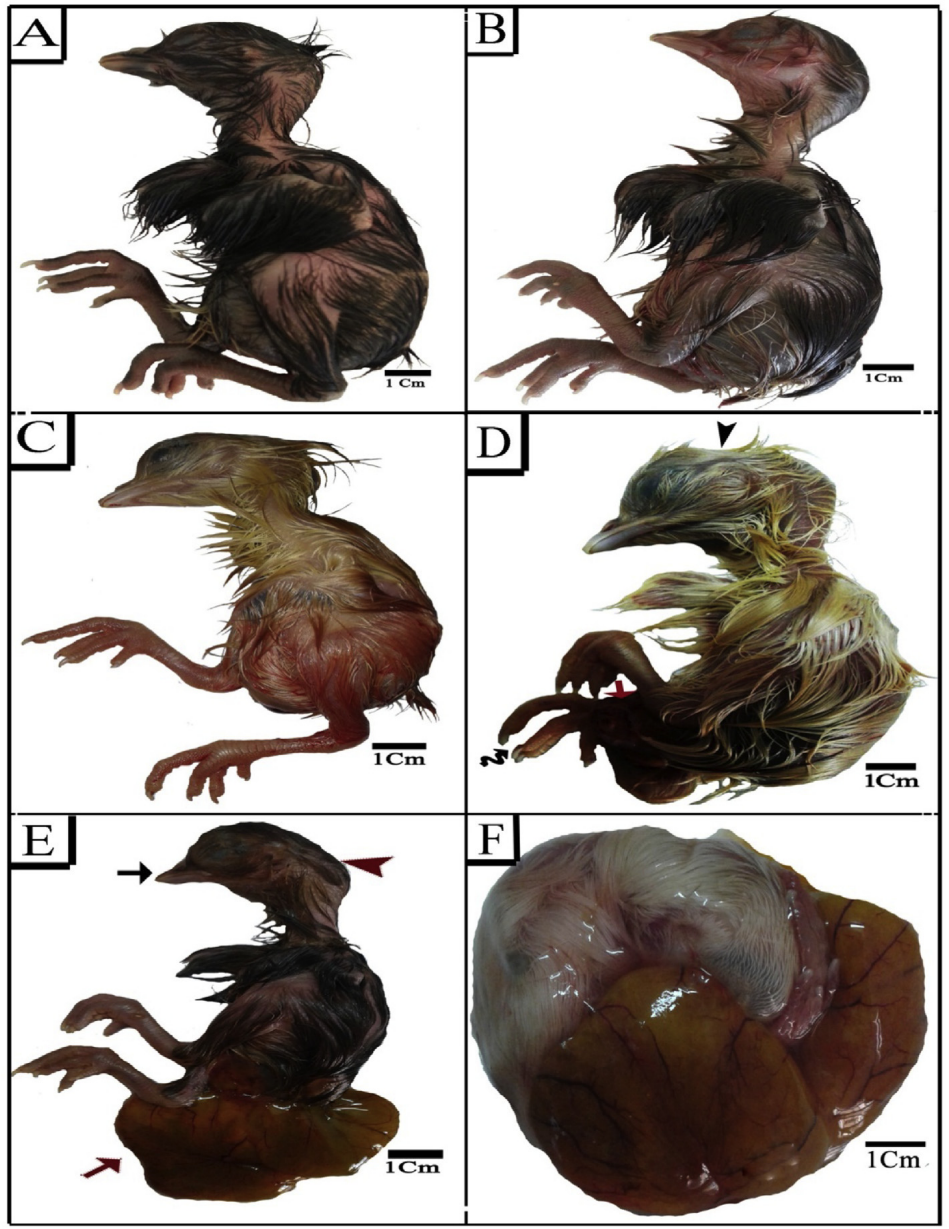
Photographs of 20-day-old chick embryos. (A) Control (B) Sham (C) Cur (D–F) SY groups showing clinodactyly (black wavy arrow), skull defects (black arrow head (, short beak (black arrow), head enlargement (red arrow head), growth retardation and omphalocele or failure retraction of yolk sac (red arrow).

The morphology of the representative control chick embryos aged 20 days is shown in (Figure 1 A). At this stage the body was covered with down feather and the head region contained the beak which became enlarged, hard and covered with horny layer as it became blunter at its tip. The nostrils were narrow slits in peak region and the external auditory aperture were narrow spherical opening with elevated edges and located at right angel of a distance about 2mm behind the eyes. The eyes were larger in size comparable to the head size and had well developed eyelids. The wing showed normal parts i.e., humerus, radius and ulna, first digit, metacarpus, second and third digits. The leg showed normal parts and covered by scales on its superior surface and cornification extend to base of toe-nail. It consisted of femur, tibia and fibula, metatarsus and the four digits which made of distinct phalanges ended by small claws where the entire planter surface of phalanges was covered with well-developed papillae. The embryos of sham and Cur injected groups showed the same normal structure like the control group (Figure 1 B&C), however some external malformations such as omphalocele and limb deformities were recorded in 14.7%, 16.6% and 13.8% for the three groups, respectively (Table 2).

**Table 2:** Percentage of morphological abnormalities of chick embryos (%) recorded in the 20^th^ day of incubation in different groups at the end of experimentation.

Malformations	Groups						
	Cont	Sh	Cur	SY	Tz	SY +Cur	Tz+Cur
Omphalocele	13.8% n=(5)	14.7% n=(5)	16.6% n=(5)	78.5% n=(11)	70% n=(14)	63.6% n=(14)	46.2% n=(12)
Limb deformities	13.8% n=(5)	14.7% n=(5)	16.6% n=(5)	35.7% n=(5)	40% n=(8)	0	0
Short beak	0	0	0	35.7% n=(5)	40% n=(8)	0	0
Scanty feather	0	0	0	0	40% n=(8)	0	0
Exencephaly	0	0	0	14.3% n=(2)	20% n=(4)	0	0
Head enlargement	0	0	0	42.8% n=(6)	50% n=(10)	18.2% n=(4)	23.1% n=(6)

The percentage of every abnormality was calculated according to each group.

**Figure 2:**
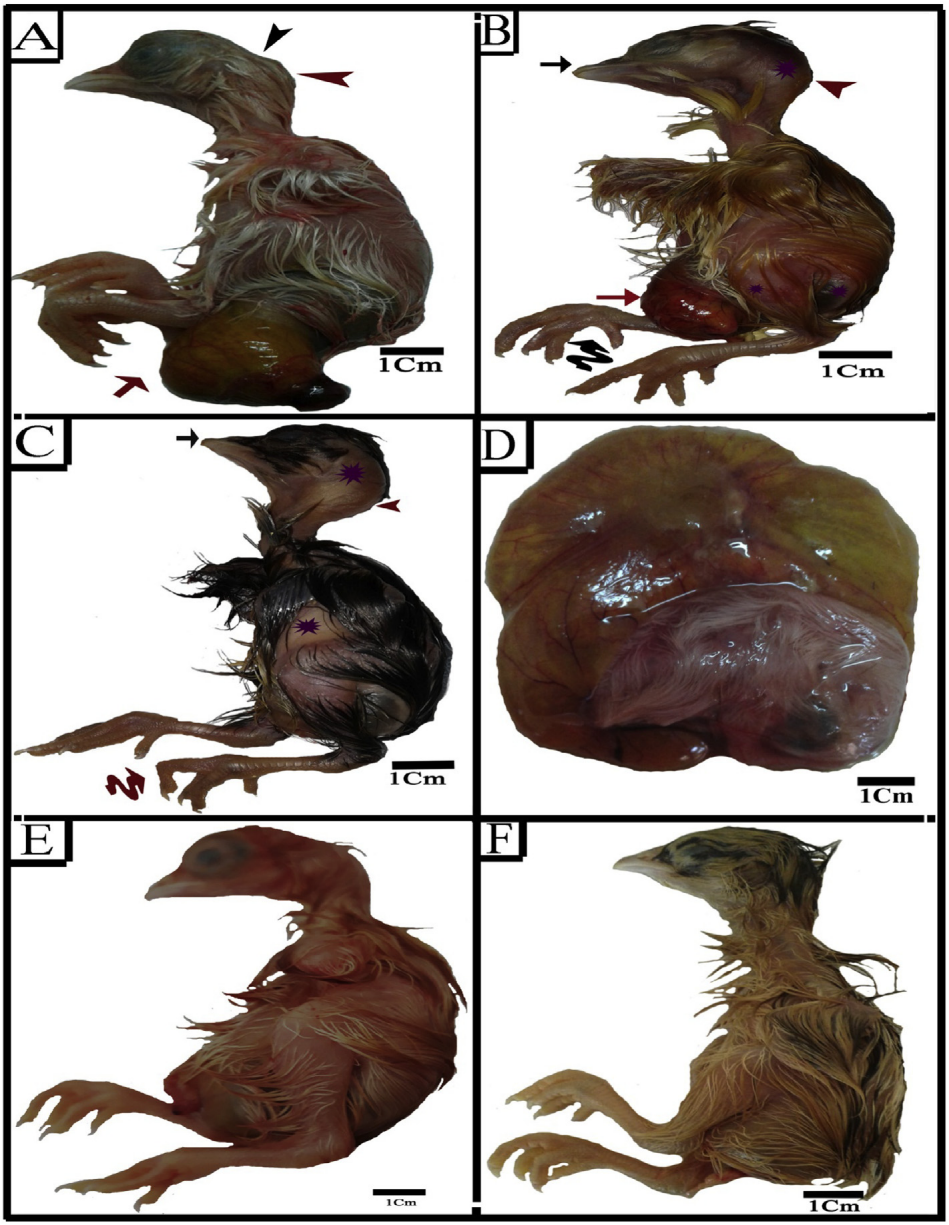
Photographs of 20 days old chick embryos. (A) SY group showing failure retraction of yolk sac (red arrow), skull defects (black arrow head) and head enlargement (red arrow head). (B–D) Tz group showing short beak (black arrow), head enlargement (red arrow head), scanty feather region (violet star), failure retraction of yolk sac (red arrow), clinodactyly (black wavy arrow), flexed limb (red wavy arrow) and growth retardation. (E) SY+Cur group (F) Tz +Cur group.

On the other hand, embryos injected with SY exhibited highly significant growth retardation and various malformations (Figure1D-F & Figure 2A) (Table 2). There was a high frequency of omphalocele, limb deformities which included clinodactyly, short beak, exencephaly and head enlargement.

Embryos injected with Tz showed similar high incidence of growth retardation and external malformations (Figure 2 B-D) (Table 2). The omphalocele, malformed limb included flexed limb and clinodactyly, short beak and scanty feather region were the most frequent malformations of this group. Moreover, 20% of embryos showed excencephaly and the head enlargement were observed in 50% of them.

Cur extract caused an evident decrease in SY and Tz induced embryo-toxicity when administrated with SY (Figure 2 E) and Tz (Figure 2 F). The chick embryos of these combined groups showed marked improvement in the shape, size and external malformations. Also, the embryos of these groups showed decrease in the degree of head enlargement (Table 2).

### 3.3 Morphometric analysis

**Figure 3:**
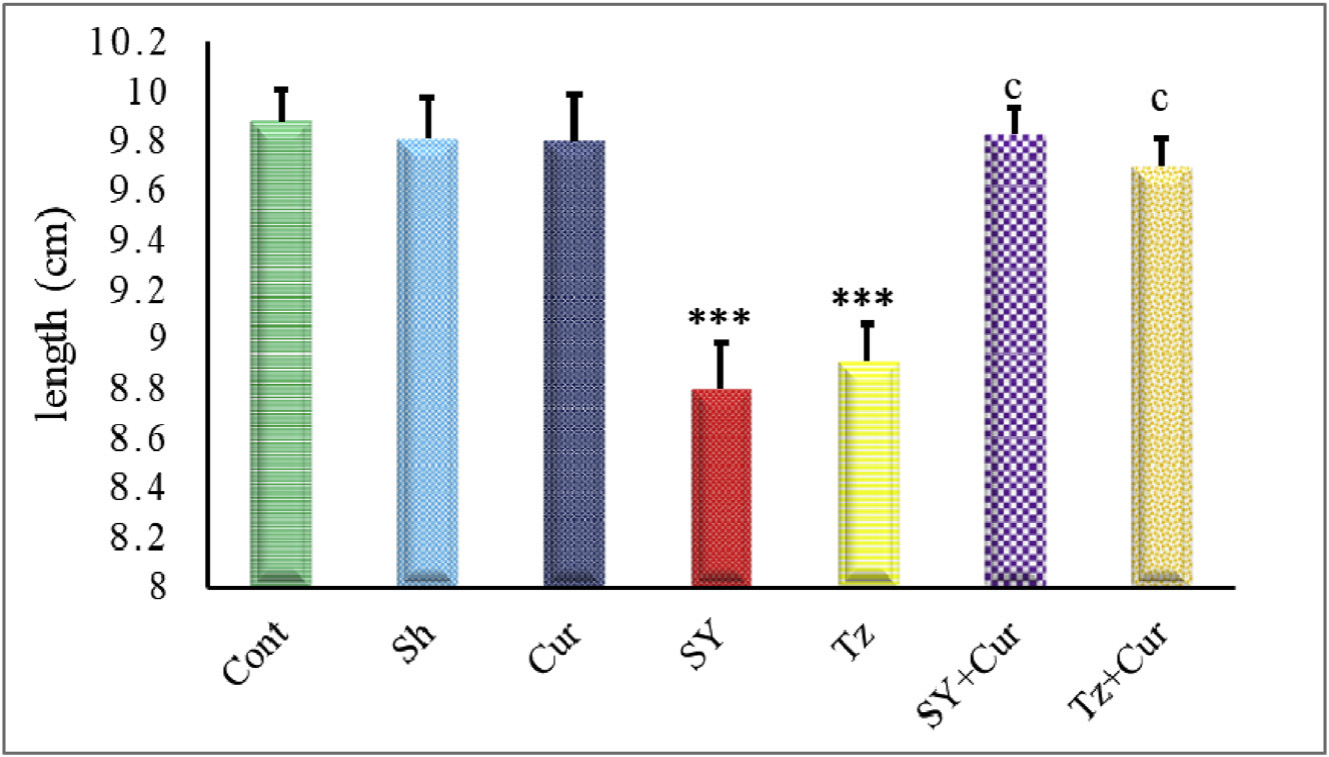
Graph showing the crown-rump length of 20-days old chick embryos of different groups. Data are represented as mean ± SEM. Asterisks (*- **- ***) refer to the P value compared with the control group. c = highly significant (p > 0.0001) compared with SY or Tz groups. ***P < 0.0001.

**Figure 4:**
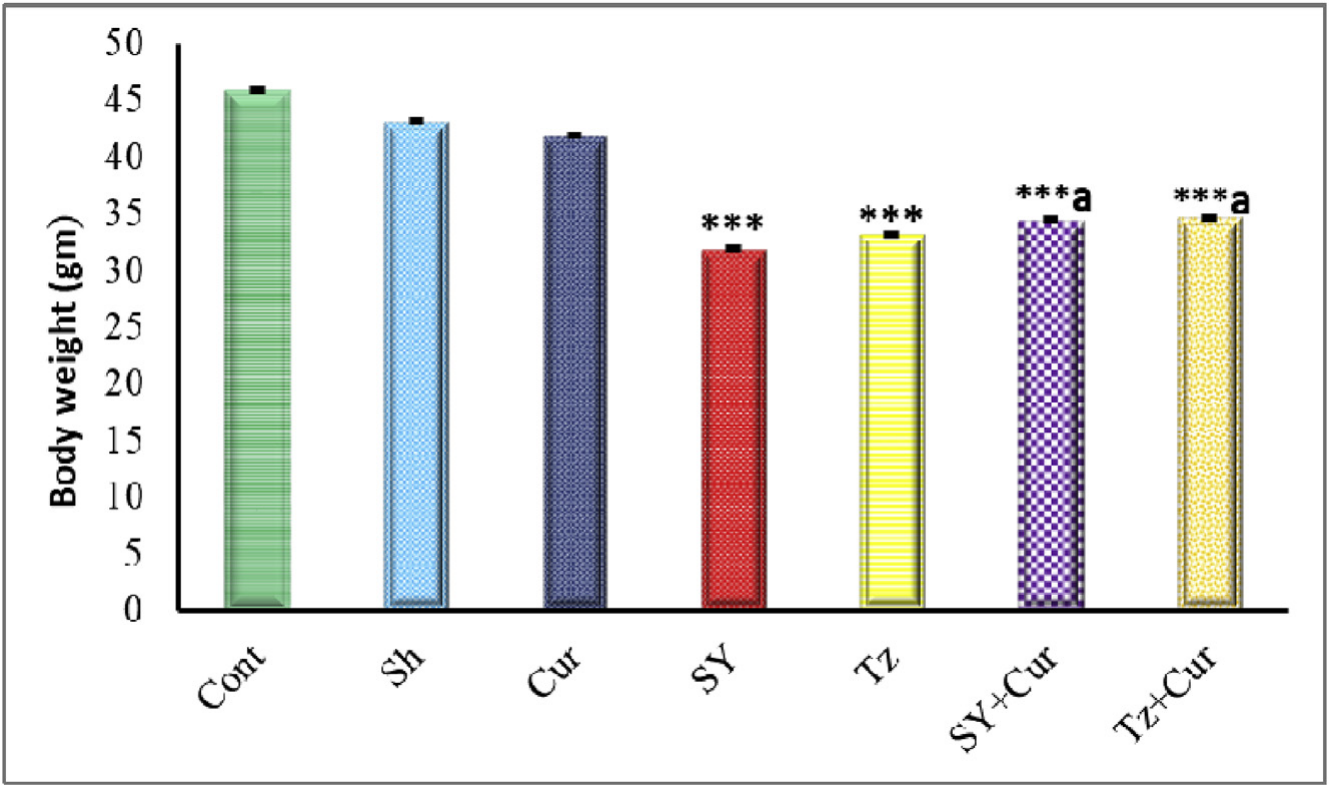
Graph showing the body weight of 20-days old chick embryos of different groups. Data are represented as mean ± SEM. Asterisks (*- **- ***) refer to the P value compared with the control group. a = slightly significant (p > 0.05) compared with SY or Tz groups. ***P < 0.0001.

The embryo-toxicity data from the control, sham, Cur, SY, Tz, Sy+Cur and Tz+Cur injected groups are presented in Figure 3 & Figure 4. The results reflected the embryo-toxicity of synthetic color on body' length and weight of the embryos. On the other hand, administration of Cur with SY and Tz led to significant ameliorations in different growth parameters.

### 3.4 Crown-rump length

Figure (3) illustrate that the embryos of control, sham and Cur injected groups had a somewhat similar values of length. On the other hand, groups injected with SY and Tz showed significant shorting compared with control group. Injection of Cur with SY and Tz caused highly significant increase in the body length compared with SY and Tz alone and insignificant difference compared with the control group.

### 3.5 Body weight

Figure (4) displayed the changes in the body weight of embryos in different groups. The embryos in control, sham and Cur injected group had similar values. There was a highly significant decrease in body weight of embryos injected with SY and Tz compared with control. Whereas, administration of Cur with SY and Tz led to clear amelioration in body weight compared with both SY and Tz alone.

### 3.6 Endo-skeletal investigation

#### 3.6.1 Control group

**Figure 5:**
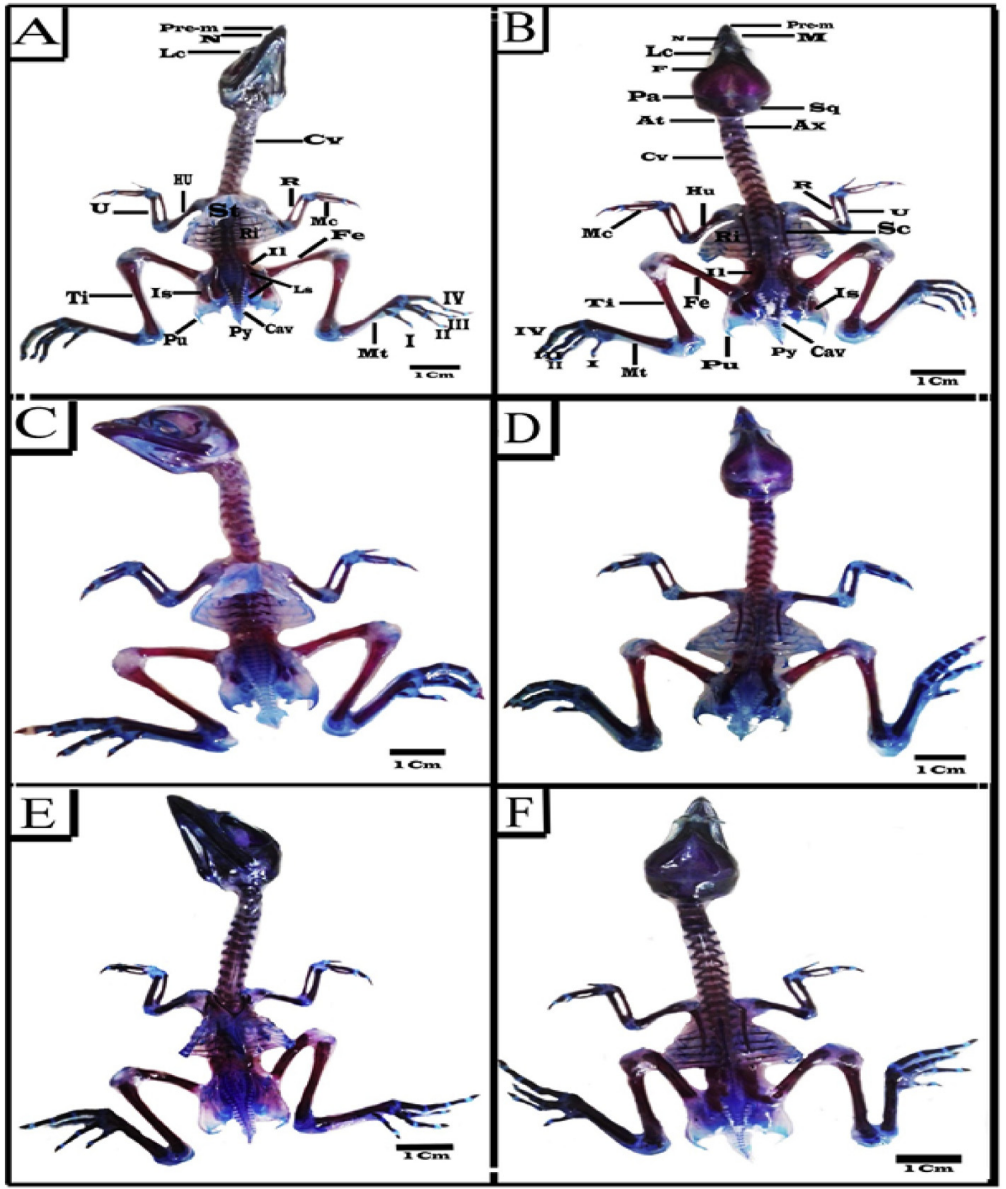
Photographs of ventral (A, C& E) and dorsal (B, D& F) views of the endo-skeletal system of 20 days old chick embryos double stained with Alizarin red S and Alcian blue. (A&B) Control (C&D) Sham (E&F) Cur group. Abbreviations: Pre-maxilla (Pre-M), Maxilla (M), Nasal (N), Lachrymal (Lc), Frontal (F), Parietal (Pa), Squamosal (Sq), Atlas (At), Axis (Ax), Cervical vertebrae (CV), Humerus (Hu), Ulna (U), Sternum (St), Radius (R), Metacarpus (Mc), Ribs (Ri), Ilium (Il), Femur (Fe), Tibia (Ti), Ischium (Is), Lumbosacral vertebrae (Ls), Pubis (Pu), Pygostyle (Py), Caudal vertebrae (Cav), Metatarsus (Mt), Scapula (Sc).

Examining the double stained endo-skeletal system of the 20-days old control chick embryos showed complete ossification in most parts of skull which represented by the premaxilla, maxilla, nasal, lachremal, frontal, parietal, palatine, mandible, pterygoid, supraoccipital, basioccipital, exoccipital and quadrate bones were stained heavy red reflect a large degree of ossification. However, squamosal, interorbital, otic and nasal capsules were less ossified. The bones of the upper jaw were slightly longer than those of lower jaw (Figure 5A&B).

The vertebral column of the embryos had normal drooping without any lateral ruckle. It was made of 42 vertebrae (14 cervical, 7 thoracic, 7 lumber, 7 sacral and 7 free caudal followed by pygostyle). The cervical vertebrae showed partial ossification at their centra and their transverse processes which stained heavy red. In the thorax, the first two and the seventh thoracic vertebrae were free, while the vertebrae 3–6 were fused. The centra of the thoracic vertebrae were mostly ossified and fused with the middle parts of the vertebral portions of the ribs. The lumber region consisted of 7 fused vertebrae while the external margins of the sacral transverse processes were fused forming a complete oval ring. The lumber and sacral regions forming synsacrum. A rostra-caudal gradient was seen with the cervical vertebrae almost completely red, while the lower lumber and sacral vertebrae still contained extensive blue staining regions and the lowest fused four to five vertebrae stained only blue. Intervertebral discs, caudal vertebrae and pygostyle were mostly cartilaginous and therefore stained blue. In some cases, however, the first two-three caudal vertebrae were partially ossified (Figure 5A&B).

The sternum was keel like and enlarged forming the carina and it was fully cartilaginous (Figure 5A). There were seven pairs of ribs originating on the thoracic vertebrae and the first, second (and sometimes the seventh) didn't reach the sternum. The third to the sixth had two segments: Vertebral segment and Sternal segment. All except the first and last had uncinate processes that project backward over the outer surface of the next rib and connected to it by a ligament. The sternal portions of the ribs connected to the sternum were cartilaginous with no evident signs of ossification (Figure 5A), while those articulating with the thoracic vertebrae were mostly ossified (Figure 5B). The vertebral ribs were well separated from each other. The scapula was narrow, thin and slightly curved shape and its caudal end didn't reach the ilium. The coracoid was the strongest bone of the shoulder girdle which articulates with the sternum from one end. Both scapula and coracoid stained heavy red, while their ends were fully cartilaginous (Figure 5B). The ischium and ilium were mostly ossified and stained heavy red, while the pubis was a narrow strip of bone that runs along the border of the ischeum to which it is joined for a short distance only and was mostly cartilaginous in nature, except in its middle part which was ossified and slightly curved (Figure 5A).

The long bones were completely cartilaginous on its epiphyses and joints. However, long bones like humerus, radius and ulna, femur and tibiotarsus, carpo-metacarpus and tarso-metatarsus were completely ossified except in the joint regions. Also most of phalanges of fore limb and hind limb were completely ossified (Figure 5A&B).

#### 3.6.2 Sham and Cur groups

Comparing the endo-skeletal structure of chick embryos of the sham and Cur groups with the control group revealed no significant difference. The skull, vertebral column, sternum, ribs and limbs showed normal skeletal structure and had the same degree of ossification like that of the control group (Figure 5C-F). Even though, 10% of the two groups respectively, had hind limb malformation in the form of flexed limb (Table 3).

**Table 3:** Endo-skeletal abnormalities in the 20 days old chick embryos of different groups (%).

Endo-skeletal abnormalities	Groups n=(10)						
	Cont	Sh	Cur	SY	Tz	SY+Cur	Tz+Cur
**Skull and Jaw abnormalities**	0	0	0	40%	50%	0	0
**Vertebral column** (Kinked tail and pygostyle)	0	0	0	10%	10%	0	0
**Ribs** (Incomplete ossification)	0	0	0	30%	40%	20%	30%
**Scapula** (Malformations)	0	0	0	0	20%	0	0
**Scapula** (Incomplete ossification)	0	0	0	10%	10%	0	0
**Ilium &Ischium** (Incomplete ossification)	0	0	0	50%	60%	40%	30%
**Hind limb** (Malformation and Incomplete ossification)	0 0	10% 0	10% 0	30% 30%	20% 40%	0 20%	0 30%

The percentage of every abnormality was calculated according to each group.

#### 3.6.3 Synthetic coloring agents groups

**Figure 6:**
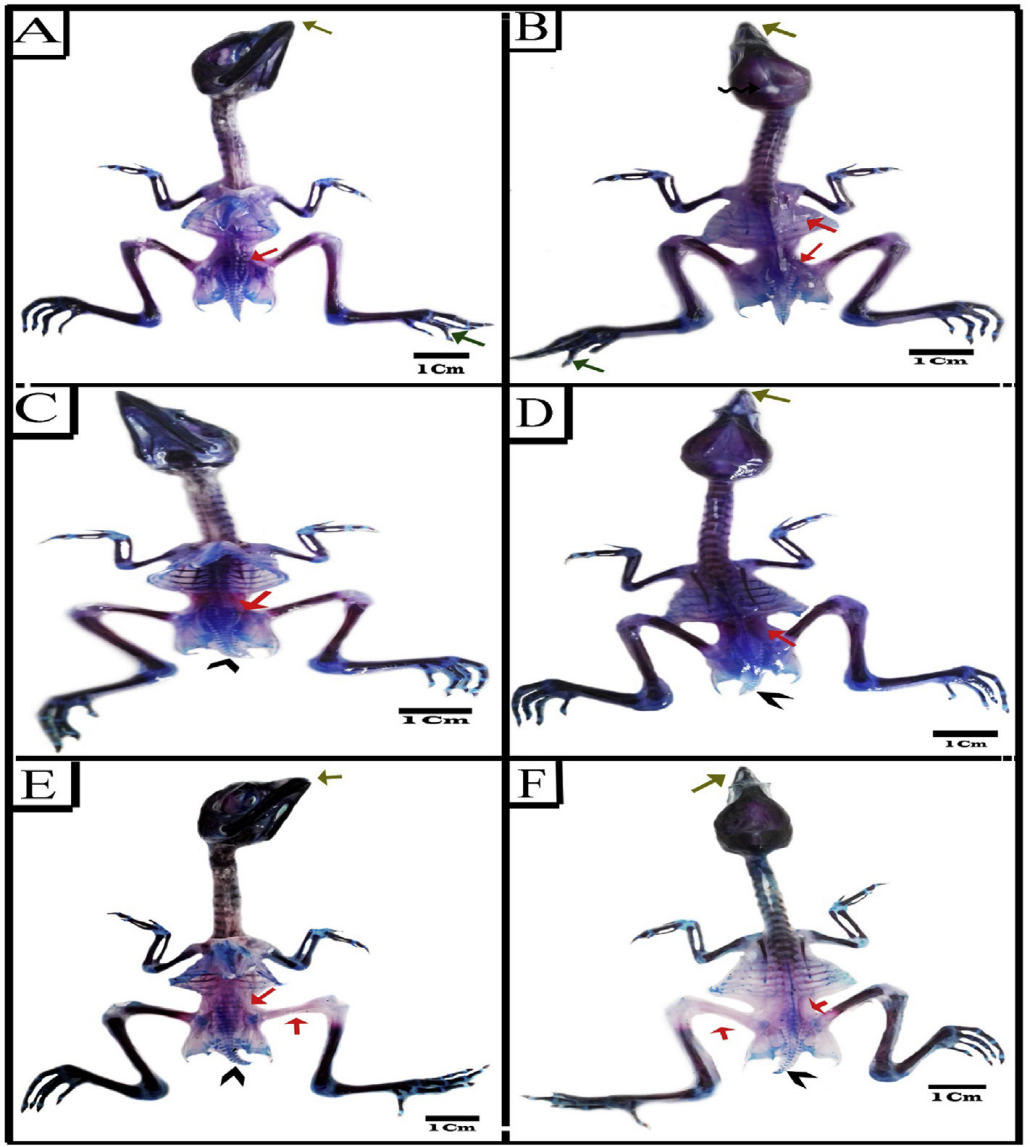
Photographs of ventral (A, C& E) and dorsal (B, D& F) views of the endo-skeletal system of representatives 20 days old chick embryos double stained with Alizarin red S and Alcian blue. (A–D) SY group showing absences of ossification pattern in some bones of pelvic girdle (orange arrow), flexed toe (green arrow), loss of parts of skull cap (wavy arrow), incomplete ossification of ribs, scapula, ischium and ilium and partial ossification of femur bone (orange arrow), short beak (olive arrow), flexed toe (green arrow), kinked tail and pygostyle (arrow head). (E&F) Tz group showing short beak (olive arrow), less ossification ossifications in pelvic girdle, femur bone, some ribs end, scapula, ischium and ilium (orange arrow), Kinked tail and pygostyle (arrow head).

**Figure 7:**
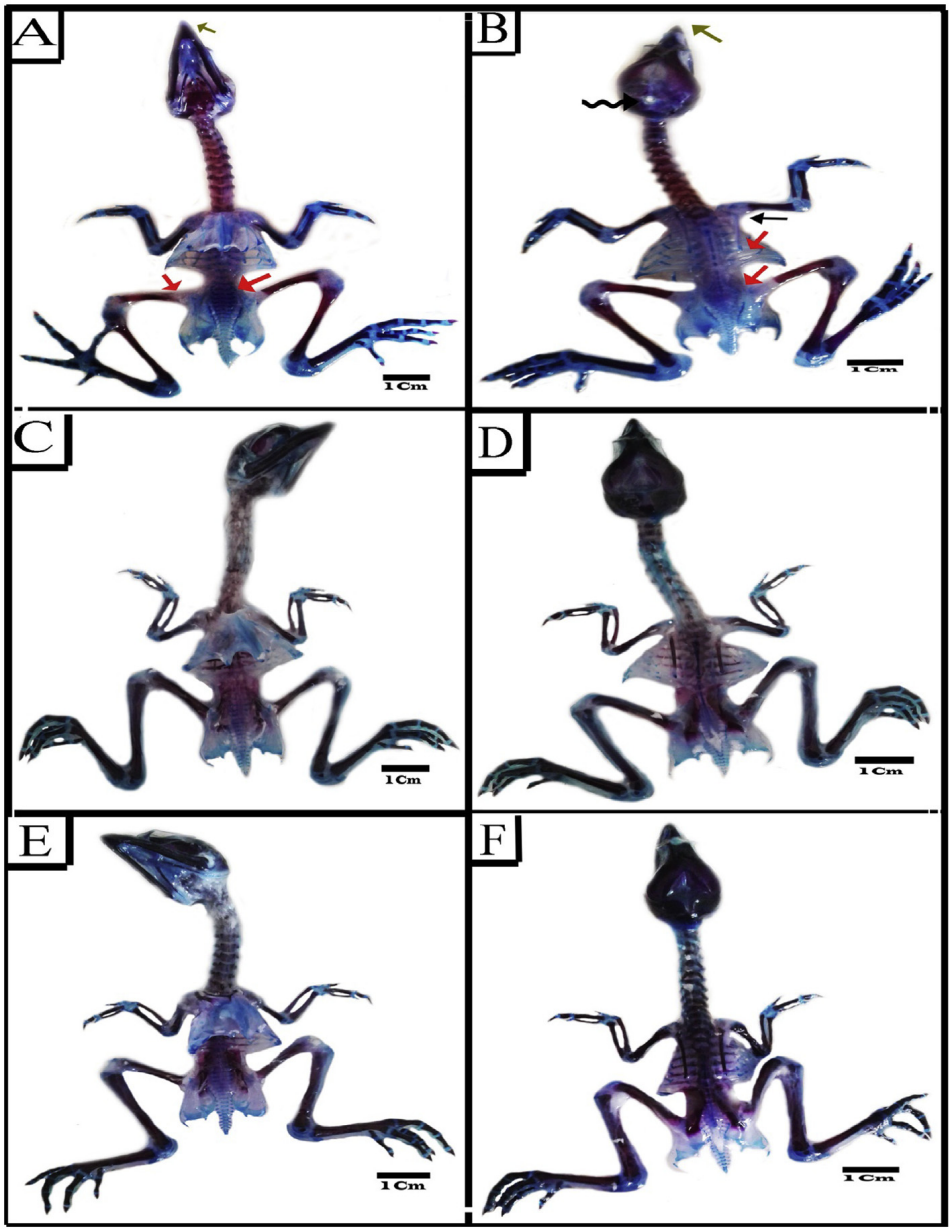
Photographs of ventral (A, C& E) and dorsal (B, D& F) views of the endo-skeletal system of representatives 20 days old chick embryos double stained with Alizarin red S and Alcian blue. (A&b) Tz group showing incomplete ossification of ischium and ilium, short beak (olive arrow), loss of parts of skull cap (wavy arrow), incomplete ossification of femur bone, ribs, scapula, ischium and ilium (orange arrow) and curved scapula (black arrow). (C&D) SY+Cur (E&F) Tz+Cur.

There were skeletal malformations in embryos injected individually with SY and Tz during the organogenesis phase of the emberyonic development (Table 3; Figure 6A-F; Figure 7A&B). Malformation of the axial endoskeleton included those of the skull cap, beak, vertebral column, strnum and ribs. The ossification pattern of embryos during organogenesis mostly similar to its counterpart of the control group. Although, there were some embryos with retarded ossification, where few bones stained red (Figure 6B-F). The skull defects were observed in losing some parts of bone especially in frontal (Figure 6B; Figure 7B). The shorting of jaws was also the main endo-skeletal abnormality especially the pre-maxilla in the upper jaw (Figure 6B-F; Figure 7A&B).

The Kinked tail and pygostyle was a vertebral defect especially in caudal vertebrae noticed in embryos with incidence of 10% (Table 3) in the two groups (Figure 6D-F). At the level of ribs, there was poor ossification in the vertebral portion with incidence of 30% and 40% of the two groups respectively (Table 3; Figure 6B; Figure 7B). In addition, the scapula of 10% embryos injected with SY and Tz showed no ossification (Table 3; Figure 6B; Figures 7B) and 20% of Tz injected embryos had curved scapula (Table 3; Figure 7B). Among the defects, came the retardation of the pelvic girdle ossification in incidence with 50% and 60% for the two groups respectively (Table 3; Figure 6A-F; Figure 7A&B). The hind limb malformation included the flexed limb (30%and 20% for the two groups respectively) and no ossification was observed in the femur of 30% &40 % of embryos (Table 3; Figure 6B&F).

#### 3.6.4 Coloring agents + Cur groups

Administration of SY and Tz followed by Cur during the organogenesis phase of the embryonic development displayed an evident decrease in the skeletal malformation compared with SYand Tz groups. The chick embryos of the combined groups showed marked improvement in the skull anomalies. Kinked tail and pygostyle anomalies disappeared after administration of Cur. Also, the incomplete ossification of ribs showed improvement and were decreased to 20% and 30% for the two groups respectively, comparing with SY and Tz. In addition, there were a significant improvement in the ossification and deformities of pelvic girdle, hind limb and scapula (Table 3; Figure 7C-F).

**Figure 8:**
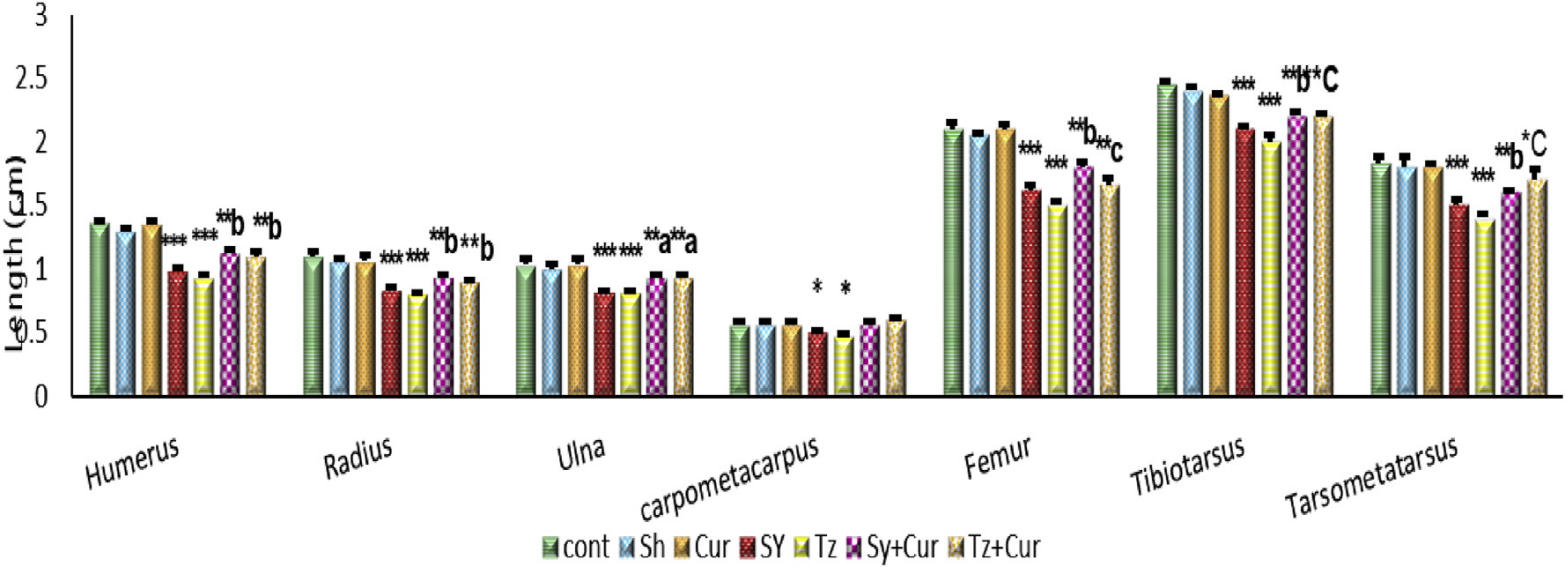
Graph showing the lengths of ossified centers of long bones in 20 days old chick embryos of different groups. Data are represented as mean ± SEM. Asterisks (*- **- ***) refer to the P value compared with the Control group. ***P < 0.0001 **P < 0.001 *P < 0.05. a = slightly significant (p > 0.05) compared with SY or Tz groups. b = moderate significant (p > 0.001) compared with SY or Tz groups. c = highly significant (p > 0.0001) compared with SY or Tz groups.

Investigation of the pattern of ossification centers of long bones revealed variations in their length among different groups which were demonstrated in Figure 8. The humerus showed insignificant difference between the control, sham and Cur groups, however, SY and Tz showed highly significant decrease compared with control group. Co-administration of Cur with SY and Tz illustrated significant increase in the length of ossification centers in humerus when compared with the control and SY, Tz groups respectively. The same pattern was seen in radius and ulna but, Carpo-metacarpus showed low significant difference in SY and Tz groups compared with control, while the combined groups showed insignificant difference with control and significant increase compared with SY and Tz groups. As for the hind limb, the ossification centers of the three long bones femur, tibiotarsus and tarso-metatarsus, were recorded in significant difference between the control, sham and Cur in the length of the three bones, femur, tibiotarsus and tarso-metatarsus. On the other hand the SY and Tz injected groups showed highly significant decrease in the length of the three bones when compared with the control group. In SY+ Cur injected group, the femur showed significant decrease when compared with the control group and significant increase when compared with SY group. The same was noticed with tibiotarsus and tarso-metatarsus. Moreover, in Tz + Cur group the three bones femur, tibiotarsus and tarso-metatarsus illustrates significant decrease when compared with the control group and significant increase when compared with Tz group.

## 4 Discussion

Today, safety limits and post-exposure health hazards associated with the consumption of food additives are of high concern to toxicologists, consumers and nutritionists (Abd-Elhakim et al., 2018). Consequently, a complete and accurate assessment of the toxicity of food additives is urgently needed.

Our result revealed that there was a significant difference in the mortality rate of chick embryos between the experimental synthetic coloring agents administrated group and the other groups as there was low mortality among control, sham and Cur groups. However, there was increasing rate in mortality associated with injection of SY and Tz at dose 14 times the ADI of both. According to the findings of this study, there was high percentage of omphalocele in both SY and Tz injected groups. On the other hand, there were low frequencies of omphalocel in sham and Cur groups. Limb deformities increased in both SY and Tz injected groups when compared with control and low deformities were shown in Cur treated group in the form of clinodactyly and flexed limb. Meanwhile, some embryos of the SY an Tz injected groups showed short beaks and exencephaly and this is in agreement with Al-Qudsi and Al-Jahdali (2012) who found that injection of 0.75mg monosodium glutamate (MSG)/egg before incubation was associated with different congenital malformation such as abdominal hernia in 7, 10 day treated embryo and brain deformation and beak malformation in 10 day treated embryo. Also, Al-Ghamdi, 2017a, Al-Ghamdi, 2017b showed that injection of chick embryo with MSG as a food additive caused congenital fetal form abdominal hernia, limb deformity and increasing in the peripheral area of the brain in the head. Moreover, the present study revealed that embryos injected with Tz showed reduction in development of feather in some region, in line with a study by Ismail and Sakr (2016) who found that hair loss was the most morphological symptoms in SY-gavage mice. Also, head enlargement occurred in both treated groups with SY and Tz. Many cytotoxic agents at lower doses may inhibit proliferative activity and result in reduced cell number, which play a central role in most teratogenesis (Singh et al., 2018).

The data obtained from the injection of colorants on crown-rump length and body weight revealed no change in Cur group as a natural color while, the SY and Tz treated groups showed decrease in both body weight and length when compared with control. This growth retardation may appeared due to generation of free radicals as the result of azo dye metabolism which resulted in oxidative stress and consequently metabolic disorder which, in turn, led to general losses of body mass (El-Desoky et al., 2017). These results were in agreement with Ismail and Sakr (2016) who found that oral feeding of mice with dosage 30 mg/kg b.wt daily with SY and with Cur at dosage 37 mg/kg b.wt daily for 30 days showed decrease in the percentage of body weight (-7.1%) and the impact of Cur on the body weight of mice was insignificant when compared with control. Furthermore, Dafallah et al. (2015) demonstrated that oral administration of SY and Tz at dose (10 mg/kg, 0.1 g/km b.wt for two groups respectively) to rats for 12 weeks as one dose every two days caused significant decrease in the gaining body weight of two groups but, when he used Cur as natural color at a dose of 10 mg/kg b.wt, there was a significant decrease in body weight also when comparing with control. Also, Kushwah & Bharti (2013) examined the effect of different doses of SY (100, 200, 400 mg/kg b.wt) and recorded the result on 7^th^, 14^th^, 21^th^ and 28^th^ day. He reported a significant decrease in body weight of treated rats comparing with control. Arefin et al. (2017) showed that no mortality and clinical signs among mice treated with Tz but there was growth retardation in mice treated with large dose (400 mg/kg b.wt) but lower dose (200 mg/kg b.wt) didn't affect the body weight gained. On the other hand, Gautum et al. (2010) showed that Tz caused significant increase in body weight of rats treated with two doses of Tz (0.2&0.4gm) for 30 days. Moreover, Himri et al. (2011) showed that rats treated with different doses of Tz (5, 7.5, 10 mg/kg b.wt) for 13 week had no mortality and clinical signs between treated groups when compared with control but there was evident growth retardation. Mehedi et al. (2009) studied the effect of Tz administration in drinking water in different concentration (0.1%, 1%, and 2.5%) on male rats which showed increasing in body weight especially in high concentration. El-Desoky et al. (2017) revealed that the daily administration of Tz at dose 7.5 mg/kg for 90 days to male rats has no mortality, but decrease the final body weight. Also, Boussada et al. (2017) showed that oral administration of Tz at 300 mg/kg for 30 days to male rats caused significant decrease in the body weight when compared with control. Recently, Hashem et al. (2019) showed that oral administration of Tz to pregnant rats in two doses (0.45, 4.5 mg/kg) from 6 days to 15 days of gestation recorded fetal mortality in both doses (10%, 10.5% respectively) and significantly decrease in fetal length and weight. On contrast, Elbanna et al. (2017) fed rats SY at a dose of 10 mg/kg and revealed significant increase in body weight. Also, Abd Elhalem et al. (2016) fed rats different doses of SY (161.4, 80.7, 40.35 mg/kg b.wt) at the end of experiment there was significant increase in body weight and no mortality between groups and no malformation were observed. Moreover, Hashem et al. (2011) revealed that oral administration of SY and Cur at dose 10 times the ADI from 6 day up to 15 day during gestation showed no fetal abnormalities. However, growth retardation was recorded in the two groups (3.2% and 10.3% respectively). On the other hand, Ai-Mashhedy and Fijer (2016) showed that Tz administration at single dose didn't had any toxic effect even at higher dose (6250 mg/kg b.wt) with no mortality.

According to the present study, synthetic coloring agents were found to cause evident malformations in the endoskeleton. The embryos from SY and Tz injected groups showed short beak, excencephaly, kniked tail and pygostyle, curved scapula especially in Tz injected group, and evident retardation in ossification of the endoskeleton. Also, the study showed reduction in the length of ossified parts of long bone of fore and hind limb in SY and Tz injected groups. On the other hand, Cur exhibited insignificant endo-skeletal anomalies when compared with control. This is in agreement with Hashem et al. (2019) who revealed that Tz at 0.45 and 4.5 mg/kg induced skeletal malformations in fetuses including missing coccygeal vertebrae, missing strenebrea, missing hind limbs and irregular ribs. A previous study by Hashem et al. (2011) showed similar findings in which oral administration of SY to pregnant females caused growth retardation and skeletal abnormalities in the metacarpal and metatarsal bones and caudal vertebrae.

Also, Afshar et al. (2014) revealed that administration of sodium benzoate as food additive to pregnant mice induced several skeletal defects on fetuses such as excencephaly, vertebral column deformities and limbs defects. Moreover, Güngörmüş et al. (2010) showed that treatment with tricalcium phosphate (E341) as food additive in 175 mg/kg to pregnant rats reduced the development of some bone of fetuses such as skull and fore-hind limb lengths. Dixit et al. (2014) explained the relation between LTB4 and osteoclastogenesis as LTB4 associated with its protein and receptors on macrophage and initiate calcium flux by cooperation between phospholipase and calcium release which activated channel to elevate intracellular calcium which activated osteoclast related genes and arouse osteoclastogenesis. More production of LTB4 enhanced osteoclastogenesis and exacerbation of the inflammatory medium.

The administration of Cur with SY and Tz during organogenesis of embryonic development decreased the mortality rate and improved the body mass when compared with the SY and Tz alone. It is probably that Cur improved the metabolism as it acting as a scavenger of free radical and increasing the ability of antioxidant. Similarly, El-Desoky et al. (2017) found that when rats fed daily for 90 days Tz at dose 7.5 mg/kg plus different doses of Cur 1gm/kg, 2gm/kg and 4gm/kg, body masses of rats fed mixtures of Cur and Tz were greater than those of rats fed only Tz. Also, Dafallah et al. (2015) reported that a mixture of equal amount of SY and Cur at dose 20 mg/kg b.wt for 12 weeks as the dose of SY was 10 mg/kg b.wt and Cur dose was also 10 mg/kg b.wt to rats revealed that using of Cur with SY increase the ratio of gaining body weight compering with SY only (82%, 68% for two groups respectively). In addition, El-Zawahry & Abu El Kheir (2007) showed that Cur administration with injection of gentamicin in rats could normalize the decrease in bodyweight which caused by gentamicin.

In our study, Cur improved the endoskeleton malformation and increased the ossification of whole endoskeleton with improvements in the lengths of long bones of both fore and hind limbs in embryos when compared with SY and Tz groups. Also, a previous study by Badawy et al. (2018) showed that oral administration of betamethasone and Cur to pregnant rats caused improvement in fetal growth whether shape, length and body weight. Also, it decreased the skeletal malformation and increases the ossification of whole endoskeleton when compared with the drug group. Also, Riva et al. (2017) showed that oral supplementation of 1 tablet of Meriva containing 1000mg of Cur to patients had osteopenia for 24 weeks improved several sides of bone health and may be helpful to decrease bone disorders such as low bone density as that it inhabited osteoclastic bone resorption. As that Cur can help in several molecular mechanisms related to bone homeostasis including the activation and differentiation of osteoclasts.

In **conclusion,** administration of SY and Tz during organogenesis of developing chick embryo at doses 1.575 mg/egg for SY and 0.375 mg/egg for Tz induced morphological malformations in feather, head, and limbs as well as reduction in the weight and length of embryos. Also, the treatment increased skeletal malformations in the embryos, including short beak, excencephaly, kniked tail and pygostyle, curved scapula and retardation in the degree of ossification. Administration of Cur with SY and Tz ameliorates the reversed effect of SY and Tz. We suggest that caution must be made when using SY and Tz as food additives during pregnancy.

## Declarations

### Author contribution statement

G. Badawy: Conceived and designed the experiments; Analyzed and interpreted the data; Contributed reagents, materials, analysis tools or data.

W. El-Sherif: Performed the experiments; Wrote the paper.

H. El-Borm: Conceived and designed the experiments; Analyzed and interpreted the data; Contributed reagents, materials, analysis tools or data; Wrote the paper.

M. Atallah: Analyzed and interpreted the data.

S. El-Nabi: Contributed reagents, materials, analysis tools or data.

### Funding statement

This research did not receive any specific grant from funding agencies in the public, commercial, or not-for-profit sectors.

### Competing interest statement

The authors declare no conflict of interest.

### Additional information

No additional information is available for this paper.
